# Supplementary material for: Non-Cholesterol Sterol Concentrations as Biomarkers for Cholesterol Absorption and Synthesis in Different Metabolic Disorders: A Systematic Review
Source: Nutrients. 2019 Jan 9;11(1):124. doi: 10.3390/nu11010124 (PMC6356200; doi:10.3390/nu11010124)
Supplement: Supplementary file 1 [file nutrients-11-00124-s001.pdf]

Supplementary Material

Non-Cholesterol Sterol Concentrations as Biomarkers for Cholesterol Absorption and Synthesis in Different Metabolic Disorders: A Systematic Review

Sultan Mashnafi, Jogchum Plat, Ronald P. Mensink and Sabine Baumgartner *

Department of Nutrition and Movement Sciences, NUTRIM School of Nutrition and Translational Research in Metabolism, Maastricht University Medical Center, 6200 MD Maastricht, The Netherlands; s.mashnafi@maastrichtuniversity.nl (S.M.); j.plat@maastrichtuniversity.nl (J.P.); r.mensink@maastrichtuniversity.nl (R.P.M.)

* Correspondence: sabine.baumgartner@maastrichtuniversity.nl; Tel.: +31-43-3881305

Table 1. Serum non-cholesterol sterol markers in subjects with gestational diabetes.

		Sitosterol		Campesterol		Cholestanol		Lathosterol		Desmosterol	
Miettinen, 2014 [53]	1st trimester Cases (n = 22)	0.78	=	1.59	=	1.39	=	1.54	=	0.72	=
	Controls (n = 30)	0.90		1.74		1.47		1.30		0.76	
	2nd trimester Cases (n = 22)	0.86	=	1.74	=	1.80	=	1.44	=	0.75	=
	Controls (n = 30)	0.97		1.89		1.81		1.32		0.75	
	3rd trimester Cases (n = 22)	0.85	=	1.67	=	2.62	=	1.33	=	0.82	=
	Controls (n = 30)	0.92		1.75		2.51		1.33		0.90	
	6 weeks PP Cases (n = 22)	0.85	=	1.82	=	1.49	=	1.27	=	1.10	=
	Controls (n = 30)	1.00		1.92		1.54		1.29		1.13	
	6 months PP Cases (n = 22)	0.91	=	1.87	=	1.40	=	1.55	=	0.94	=
	Controls (n = 30)	0.99		1.93		1.41		1.42		0.89	
	12 weeks PP Cases (n = 22)	0.91	=	1.86	=	1.38	=	1.37	=	0.85	=
	Controls (n = 30)	0.96		1.92		1.45		1.21		0.74	

Values are mean and expressed in µmol/mmoL cholesterol. Non-cholesterol markers are not-significantly different (=) between cases and controls.
